# Sea-Sickness: Its Cause, Nature, Symptoms, and Treatment, Derived from Experience and Strict Observation

**Published:** 1856-10

**Authors:** 


*Sea-Sickness: its Cause, Nature, Symptoms, and Treatment, derived from Experience and Strict Observation.* By M. L. Nelken, M. D., etc. New York: Stringer & Townsend. 1856.

This little brochure is rather an attempt to find some cure for sea-sickness, than to ascertain its cause. M. Nelken advises attention to the general health, the removal of costiveness or the checking of diarrhœa, the use of acid drinks for thirst, and, as a general thing, morphine for the vomiting. He prefers morphine to the carbonate of ammonia, chloroform or strychnia. As to prophylaxis, one may stay ashore, but we have no especial faith in treatment; and least of all have we any confidence in the belief that sea-sickness tends to improve the general health. We had a little personal experience on one occasion, and felt, for a week after leaving the vessel, like a school-boy after his first chew of tobacco.

